# Emerging Regulatory Mechanisms Involved in Liver Cancer Stem Cell Properties in Hepatocellular Carcinoma

**DOI:** 10.3389/fcell.2021.691410

**Published:** 2021-07-22

**Authors:** Duoduo Lv, Liyu Chen, Lingyao Du, Lingyun Zhou, Hong Tang

**Affiliations:** ^1^Center of Infectious Diseases, West China Hospital of Sichuan University, Chengdu, China; ^2^State Key Laboratory of Biotherapy and Center of Infectious Diseases, Division of Infectious Diseases, West China Hospital of Sichuan University, Chengdu, China

**Keywords:** liver cancer stem cells, niche, signaling pathways, stemness, hepatocellular carcinoma

## Abstract

Hepatocellular carcinoma (HCC) is the predominant form of primary liver cancer and one of the leading causes of cancer-related deaths worldwide. A growing body of evidence supports the hypothesis that HCC is driven by a population of cells called liver cancer stem cells (LCSCs). LCSCs have been proposed to contribute to malignant HCC progression, including promoting tumor occurrence and growth, mediating tumor metastasis, and treatment resistance, but the regulatory mechanism of LCSCs in HCC remains unclear. Understanding the signaling pathways responsible for LCSC maintenance and survival may provide opportunities to improve patient outcomes. Here, we review the current literature about the origin of LCSCs and the niche composition, describe the current evidence of signaling pathways that mediate LCSC stemness, then highlight several mechanisms that modulate LCSC properties in HCC progression, and finally, summarize the new developments in therapeutic strategies targeting LCSCs markers and regulatory pathways.

## Introduction

Hepatocellular carcinoma (HCC) is now the second most common cause of cancer mortality due to its resistance to chemotherapy, high rates of recurrence and metastasis, and poor prognosis (Bray et al., 2013; Torre et al., 2015; Sia et al., 2017). Although curative resection and liver transplantation are potential cures and there are new emerging medicines, the overall therapeutic effects of these strategies have limited efficacy in patients (El-Khoueiry et al., 2017; Zhu et al., 2018). One of the major reasons is that HCC has proved to be a heterogeneous disease (Anfuso et al., 2015). The situation was recently complicated by the discovery of cancer stem cells (CSCs) with highly dynamic characteristics that underlie cancer development and evolution (Iyer et al., 2019). Indeed, it is assumed that these contributors affect heterogeneity within tumors, leading to treatment resistance and tumor progression by affecting the stemness of cancer cells (Kreso and Dick, 2014).

CSCs have “stemness” characteristics similar to normal stem cells, including self-renewal capacity and differentiation and proliferation potential (Molofsky et al., 2004). CSCs and normal stem cells also share several important stemness signaling pathways such as Wnt/β-Catenin, Notch, nuclear factor (NF)-κB, Hedgehog (HH), and Janus kinase/signal transducer and activator of transcription proteins (JAK/STAT) (Chen et al., 2013). These signaling pathways have crucial roles in maintaining the characteristics of stem cells or regulating their differentiation during many developmental processes. However, the stemness properties of CSCs are lost during differentiation and are controlled by multiple signaling pathways that are highly dysregulated in CSCs (Lathia and Liu, 2017). Evidence indicates that aberrant interactions between different signaling pathways may represent key events involved in CSC pathogenesis (Yang et al., 2018). Intracellular signaling pathway dysregulation plays a significant role in enabling CSCs to retain stem cell properties.

The discovery of liver cancer stem cells (LCSCs) greatly enhanced our understanding of HCC development and progression. Despite these new developments, the mechanisms associated with CSC features are not clear, and the link between how CSCs maintain stemness and contribute to HCC malignancy is not known. Several studies have demonstrated how LCSCs can be differentiated from liver cancer cells by targeting the cluster of differentiation markers CD13 (Haraguchi et al., 2010), CD44 (Zhu et al., 2010), CD90 (Yang Z. F. et al., 2008), CD133 (You et al., 2010); epithelial cell adhesion molecule (EpCAM) (Jin et al., 2011); and keratin 19 (Van Haele et al., 2019). Here we review the latest findings of the LCSC niche and their stemness, further clarify the signaling pathways that maintain LCSC stemness, and discuss the relationship between these mechanisms and HCC progression.

## The Concept and Origin of LCSCs

Stem cells are defined a specific cell type with the characteristics of self-renewal, multi-directional differentiation potential, and high proliferative potential under certain circumstances (Molofsky et al., 2004). Stemness refers to the extent to which cells have these functional features. As stem cells differentiate, they gradually lose their “stemness.” Studying tumor origins has always been a topic of great interest, because the results may provide clues to improve anti-cancer treatments. In the past few decades, investigations have shown that only a few cancer cell subsets with tumor-initiating ability are the core source of tumorigenesis; these subsets were termed CSCs (Pierce, 1967; Eun et al., 2017). The concept of cancer as an abnormal stem cell disease was proposed based on the similar self-renewal abilities of cancer cells and normal stem cells (Wicha et al., 2006; Kreso and Dick, 2014).

Numerous studies have reported that CSCs exist in the context of multiple human cancers including HCC (Ma et al., 2007). Although the existence of LCSCs in HCC has been widely accepted, their origin remains controversial. One possibility is that liver cancer cells are the result of abnormal differentiation of undifferentiated stem cells or oval cells (OVCs) in the liver. There is evidence that these OVCs can abnormally differentiate into cancer cells under the actions of carcinogens. Many researchers believe that intrahepatic OVCs are the initiating cell of HCC (Baumann et al., 1999; Knight et al., 2000). Another possible source of LCSCs is adult hepatocytes/cholangiocytes transformed by mutations and dedifferentiation. He et al. (2013) successfully induced mature hepatocytes to dedifferentiate into HCC progenitor cells in a mouse liver cancer model by stimulating damaged liver cells with interleukin (IL-6). Nikolaou et al. (2015) used a PR-SET7-deleted experimental mouse model of liver cancer to show that promoting hepatocyte microtubule expansion can facilitate the transformation of normal liver cells into liver cancer cells. These results strongly suggest that it is entirely possible for differentiated cells to regain their stem cell status.

To better understand LCSCs, distinct markers have been reported such as CD133, CD90, CD44, CD24, CD13, OV6, Delta-like 1 homolog, and EpCAM, as well as measuring Hoechst dye efflux or aldehyde dehydrogenase activities; some of these may functionally support the LCSC phenotype including highly aggressive properties and chemoresistance (Yamashita and Wang, 2013; Xiao et al., 2017). For example, studies have indicated that EpCAM is involved in the Wnt/β-catenin signaling pathway, in which activated proto-oncogenic proteins cyclin A/E and c-Myc lead to tumorigenesis (Maetzel et al., 2009). Yang et al. (Maetzel et al., 2009; Sukowati et al., 2013) found high CD90 expression during tumor formation and reported that CD90 + cells have strong proliferation and drug resistance. Additionally, CD133 + EpCAM + Huh7 cells have powerful self-renewal, multi-directional differentiation, and clonal colony-forming capabilities (Sukowati et al., 2013). OV6 + HCC cells are more carcinogenic and resistant to chemotherapy than OV6- cells. Therefore, most LCSC makers can facilitate CSC functions in the liver and continuously maintain CSC stemness. LCSCs are highly heterogeneous and may exhibit different phenotypes in terms of carcinogenic/metastatic characteristics and chemosensitivity when purified utilizing distinct CSC markers (Yamashita et al., 2013). LCSCs were shown to be responsible for HCC metastasis and tumor recurrence, and they have innate resistance to multiple chemotherapeutic agents (Wicha et al., 2006; Adorno-Cruz et al., 2015).

## The LCSC Niche

The tumor niche consists of numerous extracellular matrix (ECM) components; cytokines; and many cell types including fibroblasts, endothelial cells, immune cells and cancer cells that can regulate the CSC fate during tumor development. Great progress has been made in the identification of potential LCSCs niches over the past few years. Various components in the microenvironment can maintain LCSC stemness through altering signaling pathways or disrupting the master transcriptional regulation factors that maintain embryonic stem cell self-renewal, such as Nanog, Oct4, and Sox2 (Shan et al., 2012; Yamashita and Wang, 2013). The ECM plays a key role in cancer progression by providing structural and biochemical support for CSCs and binds with many kinds of growth factors that interact with CSCs. For instance, one study used hyaluronic acid-based multilayer film mimic niche to select and enrich LCSCs, which showed excellent colony forming ability, and the number of CD133/CD44 double-positive LCSCs was significantly increased (Lee et al., 2015).

Many groups have demonstrated that CSCs release a variety of factors into the niche that stimulate the stemness of CSCs themselves and also induce cancer angiogenesis and the recruitment of immune cells (e.g., macrophages, dendritic cells, and T cells) and other tumor stromal cells that secrete additional factors to promote tumor progression and chemotherapy resistance (Cheng et al., 2016). Tumor-associated macrophages (TAMs) have been reported to accumulate in hypoxic areas and support angiogenesis by releasing angiogenic factors isolated from the ECM, or they can promote revascularization by releasing metalloproteinase (Carmans et al., 2010). TAMs can also enhance CSC plasticity by inducing NF-κB or the transforming growth factor (TGF)-β-dependent epithelial-mesenchymal transition (EMT) process to produce tumor necrosis factor (TNF)-α or TGF-β. In HCC, TAMs have been shown to interact with CSCs (Muramatsu et al., 2013). A subsequent study found that TAMs can secrete TGF-β to enhance CSC-like characteristics and increase their invasive capability in Hepa1-6 cells (Fan et al., 2014).

## Signaling Pathways Regulating LCSCs Stemness

Many signaling pathways have been demonstrated to control normal stem cell self-renewal and participated embryonic development and differentiation, including Wnt/β-catenin, Notch, and HH; however, persistent abnormal activation of these highly conserved pathways may underlie the characteristics of LCSCs. Table 1 presents the signaling pathways related to LCSC features, which are also described in the following questions.

**TABLE 1:** Signaling pathways activated in LCSCs.

Wnt/β-catenin	Increased invasiveness	Chen et al., 2016
	Resulted in HCC with spontaneous lung metastases	Mokkapati et al., 2014
	Lead to tumor growth and chemoresistance	Ma et al., 2007; Yang W. et al., 2008; Yamashita et al., 2009
STAT3	CD24 was found to drive LCSC genesis	Lee et al., 2011
	Maintain the LCSC population	Tang et al., 2008
	Causes CD133 + enrichment in HCC	Won et al., 2015
	Connects hepatic inflammation with LCSC expansion	Wang X. et al., 2016
TGF-β	Induces EMT to increase stemness potential and migratory and invasive capacities	Malfettone et al., 2017
	Regulate LCSC self-renewal	Bagnyukova et al., 2008
	Attenuate CSC-like features	Jiang et al., 2014
Hedgehog	Increase stemness of self-renewal and tumorigenicity	Wang R. et al., 2016
	Contributes to hepatocarcinogenesis CD133 + Hepa 1–6 cells	Jeng et al., 2013
Notch	Inhibited apoptosis to facilitate the stemness characteristics of CD90 + cells	Luo et al., 2016
BMI1	Self-renewal	Luo et al., 2016
	Proliferation of LCSCs	Chiba et al., 2010; Zhang et al., 2013

### Wnt/β-Catenin Signaling

The activation of signaling pathways is responsible for embryogenesis, liver specification, and liver regeneration in a strictly controlled manner. Dysregulation of certain signaling pathways has been implicated in maintaining LCSC stemness. With regard to the Wnt/β-catenin pathway, Chen et al. (2016) found that the rapid generation of tumor spheres and high invasiveness of side population (SP) cells isolated from HCC samples depended on this signal transduction. They also showed that increased β-catenin expression results in a marked activation of Wnt/β-catenin target genes including AXIN2, DKK1, and CCND (Chen et al., 2016). Mokkapati et al. (2014) reported that β-catenin activation in LCSCs caused HCC development with spontaneous lung metastases. Wnt/β-catenin signaling is activated following nuclear translocation of the β-catenin components Shp2 (Xiang et al., 2017), c-Myc (He et al., 1998), and EpCAM (Yamashita et al., 2007). This also leads to the enrichment of CD133 + (Ma et al., 2007), EpCAM + (Yamashita et al., 2009) and OV6 + (Yang W. et al., 2008) LCSCs that contribute to tumor growth and chemoresistance.

### STAT3 Signaling

STAT3 is a transcription factor that is constitutively activated in many malignancies and plays a key role in cancer growth and metastasis (Yuan et al., 2015). The STAT3 signaling pathway of CSCs is involved in the development of HCC. The functional LCSC marker CD24 was found to drive liver CSC genesis through STAT3-mediated Nanog regulation (Lee et al., 2011). The IL-6/STAT3 signaling pathway was proposed to be related to liver inflammation and liver regeneration (Schneller and Angel, 2019), and the mechanism is to maintain the liver CSC population through interaction with the TGF-β signaling pathway (Tang et al., 2008). A recent study demonstrated that the IL6/STAT3 signaling cascade mainly causes CD133 + enrichment in liver cancer (Won et al., 2015). Emerging evidence has implicated the aberrant expression of long non-coding RNAs (lncRNAs) in various malignancies including HCC. One lncRNA downregulated in LCSCs (lnc-DILC) mediates crosstalk between TNF-α/NF-κB signaling and the autocrine IL-6/STAT3 cascade and connects hepatic inflammation with LCSC expansion (Wang X. et al., 2016). Thus, the STAT3 signaling pathway could be a mechanism regulating LCSC stemness, as well as a possible therapeutic target.

### TGF-β Signaling

TGF-β is a central regulator in chronic liver diseases including HCC (Dooley and ten Dijke, 2012). In HCC cells, TGF-β induces EMT by increasing the expression of mesenchymal genes and CD44, which enhances their stemness potential and migratory and invasive capacities (Malfettone et al., 2017). TGF-β signals can activate differentiation programs and inhibit cell cycle progression during early carcinogenesis through intermediary Smad proteins (Fausto et al., 1990). TGFβ signaling has also been related to the malignant transformation of LCSCs. Cyclin D1-mediated activation of TGF-β/Smad signaling is an important regulatory mechanism in LCSC self-renewal and stemness. The downregulation of Socs1 attenuates the effects of TGFβ signaling, leading to oncogenic STAT3 activation and malignant cell transformation (Bagnyukova et al., 2008). Recent reports pointed out that micro (mi)RNA-mediated regulation of LCSCs is related to TGF-β signaling (Chen et al., 2021). TGF-β/Smad2 signaling can attenuate CSC-like features because miR-148a inhibits this signaling pathway in several HCC cell lines including HepG2, Huh7, and MHCC97H (Jiang et al., 2014).

### Notch Signaling

The Notch signaling pathway plays an important role in stem cell self-renewal and differentiation (Reya et al., 2001). Aberrant Notch expression may influence CSC regulation and induce tumorigenesis (Patil et al., 2006). One study demonstrated that the Notch pathway stimulated the CSC characteristics of CD90 + cells (Luo et al., 2016). The underlying mechanisms are facilitation of the G1-S transition of the cell cycle and inhibition of cell apoptosis, which promote CSC properties such as self-renewal, invasion, migration, and stem cell-related gene expression (Luo et al., 2016). Another study reported that restoring expression of the tumor suppressor gene runt-related transcription factor 3 can reduce LCSCs in HCC by inhibiting Jagged1-Notch signaling (Nishina et al., 2011). Collectively, the evidence supports exploring targeting the Notch signaling pathway in tumors.

### Hedgehog Signaling

The HH pathway has a central role in embryonic development and adult tissue homeostasis, but mutations and/or aberrant activation of the pathway involved in malignancies (McMahon et al., 2003; Bai et al., 2008). Recent evidence has indicated that HH signaling significantly facilitates liver development and regeneration, and activation of the pathway may contribute to HCC growth (Eichenmuller et al., 2009). One group proposed that HH signaling facilitates hepatocarcinogenesis, primarily in CD133 + Hepa 1–6 cells that have significantly higher colony proliferation and clonogenicity (Jeng et al., 2013). Others suggested that HH signal transduction is related to tumor chemoresistance and aggressiveness. Chen et al. (2011) found that compared with well-differentiated CD133(+)/ALDH (high) or CD133(+)/EpCAM (+) cells, enhanced HH signaling activity in poorly differentiated HCC cells may be responsible for their chemical resistance and invasiveness.

### BMI1 Signaling

B cell-specific Moloney murine leukemia virus integration site 1 (BMI1) acts as an epigenetic chromatin modifier (Park et al., 2003) and plays a central role in the self-renewal of somatic stem cells including CSCs. Aberrant BMI1 expression is associated with malignant transformation and acquisition of the malignant phenotype in HCC (Sasaki et al., 2008). SP cell analysis and sorting have been successfully applied to HCC cell lines to identify a minor cell population with CSC properties and isolate stem cells (Shimano et al., 2003). BMI1 contributes to the maintenance of tumor-initiating SP cells in HCC (Chiba et al., 2008). BMI1 repression inhibits HCC growth both *in vitro* and *in vivo* and reduces LCSC proliferation (Chiba et al., 2010; Zhang et al., 2013), suggesting that it could be a novel therapeutic target for LCSC eradication.

The Hippo pathway, along with its downstream transcriptional co-activator Yes-associated protein and transcriptional co-activator with PDZ-binding motif, has a decisive role in the pathogenesis of primary liver cancer (Van Haele et al., 2019; Nguyen-Lefebvre et al., 2021). Other signaling pathways involved in liver CSC include the Ras/Raf/mitogen-activated protein kinase (MAPK), phosphoinositide 3-kinase (PI3K)/protein kinase B (Akt)/mammalian target of rapamycin (mTOR), and NF-κB signaling pathways. Deregulation of these cascades has been shown to enrich CSCs. Notably, each individual cell line usually exhibits unique activation of oncogenic pathways, and HCC is known to be associated with aberrant stemness regulation mechanisms in LCSCs. Therefore, the mechanism of regulating the dryness of LCSCs still needs to be further studied.

## Mechanisms Modulating LCSC Function in HCC

**FIGURE 1:**
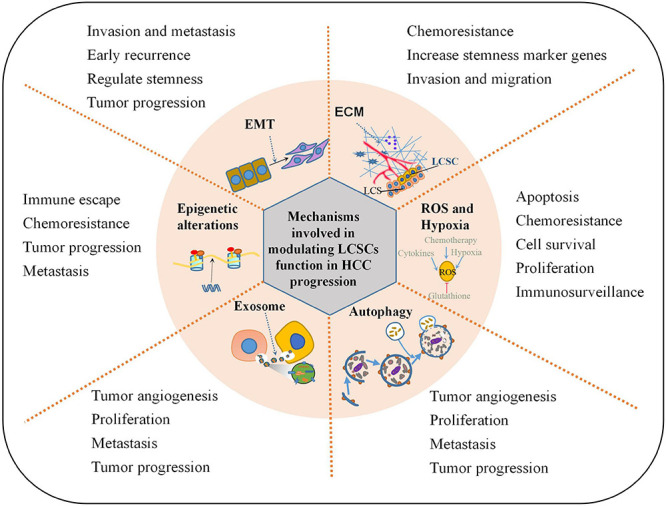
Mechanisms involved in modulating LCSCs function in HCC progression. This illustration encompasses the key mechanisms involved in regulating LCSCs properties in HCC progression: ECM alteration, EMT program induction, oxidative stress resistance, epigenetic control, autophagy modulation, secretion of extracellular vesicles or exosomes.

The difference between CSCs and normal stem cells lies in their ability to change their pluripotency and lineage-dependent differentiation. CSCs that are resistant to radiotherapy and chemotherapy can regenerate tumors after treatment ends. The current CSC theory that heterogeneous populations of HCC cells are dictated and maintained at least partially by LCSCs may help explain the process of HCC formation (Andersen et al., 2010; Lu et al., 2010). LCSCs have important roles in the initiation, maintenance, recurrence, metastasis, and resistance of HCC. Here we describe the complex mechanisms that maintain the malignant functions of LCSCs, including the ECM, EMT, exosomes, autophagy, reactive oxygen species (ROS), hypoxia, and epigenetic alterations (Figure 1).

### Extracellular Matrix-Mediated Regulation of LCSCs Properties

Accumulating evidence indicates that stem cells lose the possibility for continued self-renewal when removed from their niche, which implies an essential microenvironmental role in directing stem cell fate (Lathia and Liu, 2017). The highly dynamic ECM is a major structural component of the tumor microenvironment, and increasing evidence suggests that ECM proteins establish a physical and biochemical niche for CSCs (Nallanthighal et al., 2019). In HCC, hepatic stellate cells (HSCs) are the main source of ECM proteins in tumor stroma and greatly influence biological behaviors. For instance, HSCs may decrease hepatoma cell sensitization to chemotherapeutic agents by promoting EMT and CSC-like features *via* hepatocyte growth factor/Met signaling (Yu et al., 2013). In addition, NEDD9 is one of four members of a family of protein scaffolds that is crucial for HCC metastasis; it has been confirmed that NEDD9 downregulates Smad7 to activate Smad signaling and bind the FAK-Src-Crk complex to promote EMT and LCSC stemness (Wang et al., 2017). Furthermore, the pericellular matrix formed by hyaluronic acid and its interaction with tumor cell receptors can exacerbate malignancy and treatment resistance and aggravate the CSC phenotype by enhancing stem-cell marker genes and facilitating invasion and migration (Avnet and Cortini, 2016).

### LCSC Acquisition of Malignant Function by EMT

The EMT plays crucial roles in developmental processes and tumor invasion and metastasis (Thiery et al., 2009). E-cadherin loss and upregulation of mesenchymal markers are hallmarks of the EMT process that have been associated with invasiveness, metastatic potential, and poor clinical outcomes in several cancers including HCC (Thiery and Sleeman, 2006; van Zijl et al., 2009). Initial work showed that EMT program activation in epithelial cells induces the acquisition of stem cell characteristics that may be conducive to CSC emergence in the context of cancer (Fabregat et al., 2016). For example, an *in vitro* study demonstrated that EMT activation could induce CSC characteristics, which is mediated by hypoxia-inducible factor 1 alpha (HIF-1α)-upregulated Notch intracellular domain expression (Jing et al., 2019). Notably, tumor cells that express EMT-related proteins also express stemness-related proteins in HCC (Kim et al., 2011). According to a study by Yamanaka et al. (2018), enrichment of the functional LCSC marker CD13 was correlated with early recurrence and shorter survival in patients with HCC. In the process of EMT, the switch from CD44v to CD44s expression is very important for the regulation of CSC stemness in cancer progression (Skandalis et al., 2019). Specifically, CSC expression of epithelial splicing regulatory protein 1, a key transcription factor required to control the transition from CD44v to CD44s in EMT, can be inhibited by Zinc finger E-box binding homeobox 1 (ZEB1) (Preca et al., 2015); CD44s in turn induces ZEB1 expression to form a self-sustaining loop that further facilitates the EMT process, enabling cancer cells to acquire stemness without external stimuli (Park et al., 2016). In addition, EMT caused by the synergistic effect of CD44 and TGF-β1 is more likely show enhanced migration and lead to aggressive HCC progression (Park et al., 2016). CD44 has emerged as an LCSC marker that strongly induces EMT together with TGF-β1. Park et al. (2016) recently reported synergistic interactions between CD44 and TGF-β1 in EMT induction and CSC properties through the AKT/GSK-3β/β-catenin pathway in HCC cells. Moreover, Hu et al. (2017) found that overexpression of mammalian-enabled protein in HCC cells facilitated stem cell markers, EMT markers, and tumorigenicity through the extracellular signal-regulated kinase (ERK) and β-catenin signaling pathways. In addition, it was confirmed that Aurora Kinase A (AURKA), an oncogene involved in tumor development, can induce the EMT process and CSC properties through the PI3K/Akt pathway; silencing AURKA inhibits radiation-enhanced cell invasiveness of HCC (Chen et al., 2017). Therefore, EMT programs may be involved in the acquisition of malignant LCSC function during cancer progression.

### Effect of Oxidative Stress and Hypoxia on the Role of LCSCs in HCC Progression

One of the striking characteristics of CSCs is their ability to form a specialized niche to adapt to changing microenvironmental conditions and exploit the characteristics of self-renewal and differentiation to drive tumor growth and progression (Baumann et al., 2008). Due to their heterogeneity, CSCs exhibit distinct metabolic phenotypes in different tumor types in terms of stemness features (Baumann et al., 2008). In particular, ROS levels are intimately tied to cellular metabolic phenotype (Yeo et al., 2013). Continuous ROS accumulation can induce apoptosis of both normal cells and cancer cells (Wiseman and Halliwell, 1996; Yamamori et al., 2012). Interestingly, CSCs can limit ROS production to maintain stem cell characteristics, inducing dormancy and enhancing drug resistance (Carnero et al., 2016; Clark and Palle, 2016). Several studies suggest that CSCs preferentially depend on glycolytic pathways, which have low or absent rates of oxidative phosphorylation and high lactate production (Wong et al., 2017; Chang et al., 2018). CSCs can also increase ATP production rates and reduce ROS production due to the Warburg effect in response to stressful environmental conditions characterized by low oxygen (hypoxia) (Wong et al., 2017; Chang et al., 2018). Interestingly, Chang et al. (2018) found that ROS-independent endoplasmic reticulum stress mediates nuclear factor erythroid 2-related factor 2 (NRF2) activation to promotes the Warburg effect and maintain CSC stemness-associated properties. Another study suggested that the hyaluronan-CD44 axis can upregulate p62 to deactivate KEAP1, which promotes NRF2 activation and the subsequent transcription of antioxidant response genes to inhibit ROS accumulation and induce drug resistance in cancer cells. In addition, CD44v9 has been reported to be associated with NRF2 activation and poor overall survival of HCC patients (Kakehashi et al., 2016).

Under hypoxic conditions, cells suppress energy-intensive mRNA translation by modulating the mTOR and pancreatic eIF2alpha kinase (PERK) pathways. Skandalis et al. (2019) found that hypoxic ROS regulate mTOR and PERK to control mRNA translation and cell survival. A recent study demonstrated that hypoxic CSCs impede CD8 + T cell proliferation and activation and inhibit immunosurveillance (Wei et al., 2011). Hypoxia also protects CSCs from chemo- and radiotherapy, and oxidative stress plays a central role in maintaining CSC stemness under hypoxia. Remarkably, hypoxia can promote CSC survival and EMT through ROS-activated stress response pathways (Liu et al., 2008) and ROS-induced TGF-β and TNF-α signaling pathways (Pavlides et al., 2010).

### Epigenetic Alterations Provide LCSCs With a Survival Advantage

Epigenetic mechanisms including DNA methylation, post-translational histone modifications, chromatin conformation changes, and miRNA expression are the keys to normal stem cell differentiation. Similar processes have been shown to enable cancer cells to restore stem cell-specific characteristics (Baumann et al., 2008). Dysregulation of epigenetic mechanisms such as DNA methylation leads to abnormal epigenetic alterations that can contribute to CSC progression (Lathia and Liu, 2017). Specifically, DDX3 reduction can inhibit tumor-suppressive miRNA expression, promote up-regulation of DNA methyltransferase 3A (DNMT3A), enrich DNMT3A binding on promoters of tumor-suppressive miRNAs, and cause hypermethylation (Lathia and Liu, 2017). In addition, arsenic trioxide can enhance sensitivity to chemotherapy *via* the NF-κB pathway, which modulates de-methylation of miR-148a and inhibits LCSC properties (Wang et al., 2020). Assessing DNA methylation patterns also could provide a new approach to determining the origin of recurrent HCC (Zhang et al., 2015). Histone acetylation is another epigenetic mechanism that plays an important role in CSC regulation. NF-κB-mediated inhibition of histone deacetylases (HDACs), which are chromatin-remodeling enzymes, can facilitate an effective IKK inhibitor that targets a selected subgroup of CSCs in human HCC cell lines (Marquardt et al., 2015). HDAC inhibitors are useful for eradicating Spalt-like transcription factor 4 (SALL4)-positive HCC cells through their inhibitory effects on histone deacetylation *via* the nucleosome remodeling and deacetylation complex (Marquardt and Thorgeirsson, 2013). Encouragingly, a recent study demonstrated that SALL4 plays a role in controlling HDAC activity and contributing to the maintenance of HCC with stem cell features (Zeng et al., 2014). Moreover, BORIS, a testes-specific CTCF paralog (CCCTC-binding factor-like), up-regulates Oct4 *via* histone methylation to facilitate CSC-like characteristics in HCC cells (Zeng et al., 2014). miRNAs are another epigenetic mechanism responsible for regulating CSCs. For example, DDX3, a member of the DEAD-box RNA helicase family (Schroder, 2010), represses stemness in HCC by epigenetically modulating tumor-suppressive miRNAs (including miR-200b, miR-200c, miR-122, and miR-145) (Li H. K. et al., 2016). Jiang et al. discovered that miR-500a-3p promotes LCSC characteristics including enhanced spheroid formation, increased SP fraction (purified from HCC cells harbors CSC-like properties), and upregulated expression of CSC factors (Zhang et al., 2017). Whole transcriptomic analyses of SP cells can generate common SP-gene expression profiles for predicting the clinical outcome (survival and recurrence) of in-patients with HCC (Marquardt et al., 2010). Epigenetic modification of miR-429 can manipulate LCSCs by targeting the RBBP4/E2F1/Oct4 axis, suggesting that targeting miR-429 might inactivate LCSCs, thus providing a novel strategy for HCC prevention and treatment (Li et al., 2015).

### HCC Tumorigenesis and Progression Regulated by LCSC-Derived Extracellular Vesicles

Extracellular vectors (EVs) derived from CSCs (including exosomes, microvesicles, and apoptotic bodies) are important mediators that modulate communication between CSCs and their niches. EVs are rich in enzymes, miRNAs, transcription factors, heat shock proteins, major histocompatibility complexes, cytoskeleton components, signal transducers, and ECM effectors (Milane et al., 2015) that mediate the exchange of intracellular components and affect tumor aggressiveness. Emerging evidence indicates that CSCs and TAMs promote HCC tumorigenesis and progression. A functional study revealed that treating HCC cells with TAM exosomes or transfecting them with miR-125a/b suppressed cell proliferation and stem cell properties by targeting CD90, a marker of HCC stem cells (Wang et al., 2019a). Tumor angiogenesis has also been associated with exosomal export of specific RNA species in CSCs. For example, CD90 + liver CSC-released exosomes stimulated angiogenesis *via* the lncRNA H19, which mediated increased expression of vascular endothelial growth factor (VEGF) receptor 1 in endothelial cells (Conigliaro et al., 2015). Mechanically, Cheng et al. (2019a) showed that p120ctn in exosomes secreted from liver cancer cells suppresses HCC cell proliferation and metastasis and LCSC expansion *via* the STAT3 pathway. Another publication described the impact of CSC-derived exosomes on HCC progression *in vivo*. Specifically, CSC-exosomes can reduce apoptosis (marked by downregulation of Bax and p53, upregulation of Bcl2, and increased proliferating cell nuclear antigen immunostaining), increase angiogenic activity (shown by up-regulation of VEGF), enhance metastasis and invasiveness (indicated by upregulation of PI3K and ERK proteins and their downstream target matrix metalloproteinase 9 and downregulation of tissue inhibitor of metalloproteinase-1), and induce EMT (marked by increased serum and hepatic levels of TGFβ1 mRNA and protein) (Alzahrani et al., 2018).

### Autophagy in LCSCs Maintenance

Following the establishment of the CSC theory and the discovery of LCSCs in HCC, autophagy was proposed as a vital mechanism driving cell fate (Nazio et al., 2019). Remarkably, in addition to maintaining cellular homeostasis, autophagy also affects cellular processes such as EMT and migration, both of which drive tumor progression and metastasis (Kiyono et al., 2009; Qiang et al., 2014; Sharifi et al., 2016). Indeed, the accelerated carcinogenesis observed in autophagy-deficient murine models strongly supports the hypothesis that autophagy prevents malignant transformation (Cianfanelli et al., 2015; Mainz and Rosenfeldt, 2018). Hypoxia-induced autophagy has been shown to be essential for survival of hepatic CD133 + CSCs, which is mediated by HIF-1α (Nazio et al., 2019). Notably, Li et al. found that CD133 + LCSCs could resist interferon-γ-induced autophagy, which might also be a mechanism through which CSCs resist immune eradication (Li J. et al., 2016). Lai et al. discovered that homeobox-containing protein 1 expression in hepatocytes could protect against HCC progression, and the underlying mechanisms may include promoting autophagy, inhibiting CSC phenotype, and increasing the sensitivity of tumor cells to natural killer cell cytolysis (Zhao et al., 2018). Autophagy can also regulate CSC resistance to chemotherapy drugs. For example, transactivation response element RNA-binding protein 2 is destabilized through autophagic-lysosomal proteolysis, thereby stabilizing the protein expression of the CSC marker Nanog to facilitate multikinase inhibitor sorafenib resistance in HCC cells (Lai et al., 2019).

### Hepatitis Virus and LCSCs

Chronic infection with hepatitis B virus (HBV) has long been linked to HCC development. Studies have found that the C-terminally truncated HBx (HBx-ΔC) plays an important hepatocarcinogenesis by conferring enhanced invasiveness and reducing the apoptotic response in HCC cells (Ma et al., 2008; Yip et al., 2011; Zhu et al., 2015). Ng et al. (2016) recently found that HBx-ΔC—in particular at the 140 aa and 119 aa breakpoints—enhances stemness properties *in vitro* and induces a CD133 + LCSC subpopulation in HCC by modulating an altered genomic profile involving the FXR pathway and possibly drug metabolism. It was also previously reported that hepatitis C virus (HCV) infection of primary human hepatocytes (PHH) can induce an EMT state and extend cell lifespan (Bose et al., 2012). Compelling evidence has suggested that HCV infection of PHH induced a significant increase in the number of spheres on ultralow binding plates, and it enhanced EMT and CSC markers and tumor growth in immunodeficient mice (Kwon et al., 2015). These data support the hypothesis that hepatitis virus infection plays a crucial role in conferring stemness properties that contribute to HCC initiation and growth.

### Non-coding RNA and LCSCs

A variety of non-coding RNAs play an important role in LCSC self-renewal and promote tumor propagation. For example, a recent study showed that lncHDAC2 drives the self-renewal of LCSCs *via* activation of HH signaling (Wu et al., 2019). Another lncRNA termed HAND2-AS1 promotes LCSC self-renewal and drives liver oncogenesis (Wang et al., 2019b). The novel lncRNA THOR (testis-associated highly conserved oncogenic lncRNA) was also upregulated in liver CSCs and could promote HCC cell dedifferentiation and liver CSC expansion by targeting β-catenin signaling (Cheng et al., 2019b). Ling Qi and colleagues demonstrated that lncCAMTA1 physically associates with the calmodulin-binding transcription activator 1 (CAMTA1) promoter, induces a repressive chromatin structure, and inhibits CAMTA1 transcription, thereby promoting HCC cell proliferation, CSC-like properties, and tumorigenesis. Furthermore, deregulation of miRNA expression in cancer cells (including HCC cells) is well documented, and the involvement of miRNAs in orchestrating tumor genesis and cancer progression has been recognized. miR-26b-5p imparts metastatic properties, helps maintain Ep + CSCs *via* HSC71/HSPA8, and augments malignant features in HCC (Khosla et al., 2019). Overexpression of miR-589-5p suppressed CD90 + CSC characteristics such as Oct4, Sox2, and Nanog expression; a high likelihood of forming cell spheres; high invasiveness; and high tumorigenicity (Zhang et al., 2016). MiR-491 attenuates CSC-like properties of HCC by inhibition of GIT-1/NF-κB-mediated EMT (Yang et al., 2016). MiR-452 directly acts on Sox7 that physically binds to β-catenin and TCF4 in the nucleus, then inhibits Wnt/β-catenin signaling and promotes the stem-like characteristics of HCC (Zheng et al., 2016). Given the significant contribution of non-coding RNA to LCSC properties, targeting specific non-coding RNA clusters might be an effective HCC treatment.

## Therapeutic Strategies to Target LCSCs Pathways

Currently, targeting LCSCs and/or eradicating LCSCs brings hope of curing HCC. We have known that a variety of signaling pathways including STAT3, TGF-β, Hedgehog (Hh), Notch, Wnt and BMI1 are involved in the renewal of normal stem cells and the maintenance of tissue homeostasis. Dysregulation of these pathways is believed to be involved in driving CSC activity in a variety of cancers (including HCC) through different mechanisms (Gu et al., 2020; Huang et al., 2020; Babaei et al., 2021). More specific inhibitors of signaling pathways are currently under development and investigation, which may be potential therapeutic agent for eradicating liver CSCs and overcoming chemotherapy resistance of HCC. For example, A study demonstrated that Salinomycin significantly reduces the tumorigenicity of LCSCs *in vivo* by suppressing the Wnt/β-catenin signaling pathway 31971116. A novel small-molecule Wnt inhibitor, IC-2, has the potential to suppress LCSCs that may *via* inhibition of the CBP–β-catenin complex formation (Huang et al., 2020). Reportedly, aminopeptidase N (APN, also known as CD13) is a marker of semi-quiescent CSC. Hedgehog pathway molecules was altered, including upregulated S Hedgehog expression and downregulated smoothened expression in tumor fractions after GDC-0449 treatment, which effectively reduced tumor size and cell infiltration of the HCC in mice (Jeng et al., 2015). The CD13 inhibitor BC-02, a compound obtained by combining the CD13 inhibitor Battatin and Fluorouracil (5-FU), damages the properties of liver CSCs by targeting CD13 and up-regulating intracellular ROS and DNA damage induced by ROS (Dou et al., 2017). The treatment strategies for signaling pathways or mechanisms are summarized in Table 2.

**TABLE 2:** Summary of various agents in research development to target LCSCs pathways.

**Target**	**Agent**	**Population**	**Results**	**References**
Wnt/β-catenin	Salinomycin	Human HCC cell lines	Reduces the tumorigenicity of LCSCs	Liu et al., 2020
Wnt	IC-2	Human HCC cell lines	Suppresses liver CSC properties	Seto et al., 2017
Hedgehog	GDC-0449	Mouse hepatoma ML-1 cells	Mitigate the mice HCC growth	Jeng et al., 2015
Hedgehog	LDE225	Human HCC cell lines	Suppress EMT and migration of metastatic cells	Ding et al., 2017
Notch	RO4929097	Miouse liver progenitor cell	Reduces Tumor Growth, Tumor Malignancy and Liver Fibrosis *in vivo*	Jung et al., 2016
Notch	DAPT	Human HCC cell lines	Significantly reduced CD133/epcam positivity	Kahraman et al., 2019
TGF-β	LY2157299 (Galunisertib)	Human HCC cell lines	Suppresses the staminal phenotype in hepatocellular carcinoma by modulating CD44 expression	Rani et al., 2018
BMI1	RU-A1	Human HCC cell lines	Impair Self-Renewal and Tumorigenic Capability in HCC	Bartucci et al., 2017
NF-κB	Curcumin	Primary HCC cell line	Restrains stemness features in liver cancer	Marquardt et al., 2015
CD13	BC-02	Human HCC cell lines	Targeting CD13 and up-regulating intracellular ROS and DNA damage induced by ROS	Dou et al., 2017

## Conclusion

There is a growing body of evidence indicating that CSCs are the root cause of cancers and are responsible for metastasis, resistance to conventional treatments, and tumor recurrence; however, the molecular mechanisms underlying the potential roles of LCSCs in HCC origin and progression have not been fully elucidated. LCSC status and survival are controlled by multiple signaling pathways, and there are several mechanisms to maintain their malignant function. This overview presented findings suggesting that LCSCs drive HCC occurrence and development. In theory, if the LCSC subpopulation can be eliminated or have reduced stemness, it would be a way to control or even cure HCC. Although a great deal of effort is being made to reduce cancer stemness by targeting stemness signaling pathways, there are still many challenges given the complex biologic properties of CSCs. Perhaps the current focus should be on clarifying maintenance mechanisms involved in the malignant function of LCSCs. Since LCSCs must be eradicated to prevent HCC progression, recurrence, or metastasis, targeting the vital CSC signaling pathways is an attractive cancer treatment strategy. A comprehensive understanding of LCSC stemness and the mechanisms involved in cancer progression may help identify potential therapeutic targets and develop more effective therapies.

## Author Contributions

HT and LZ contributed to the conception of this study. DL and LC drafted the manuscript. HT, LZ, and LD revised the manuscript. All authors read and approved the final manuscript.

## Conflict of Interest

The authors declare that the research was conducted in the absence of any commercial or financial relationships that could be construed as a potential conflict of interest.
